# Real-Time Associations Between Subjective Cognitive Concerns and Daily Activity Participation in Persons Aging With Stroke: Smartphone-Based Ecological Momentary Assessment Study

**DOI:** 10.2196/80222

**Published:** 2026-07-14

**Authors:** Jiali He, Quoc Bui, Mandy W M Fong, David C Mohr, Christopher L Metts, Alex W K Wong

**Affiliations:** 1 Department of Physical Medicine and Rehabilitation, Northwestern University Feinberg School of Medicine, Chicago, IL, United States; 2 Center for Rehabilitation Outcomes Research, Shirley Ryan AbilityLab, 355 E Erie Street, Chicago, IL, 60611, United States, 1 312-238-1742, 1 312-238-4572; 3 Graduate Medical Education Consortium, South Texas Health System, Edinburg, TX, United States; 4 Michigan Avenue Neuropsychologists, Chicago, IL, United States; 5 Center for Behavioral Intervention Technologies, Northwestern University Feinberg School of Medicine, Chicago, IL, United States; 6 Department of Preventive Medicine, Northwestern University Feinberg School of Medicine, Chicago, IL, United States; 7 Department of Pathology and Laboratory Medicine, Medical University of South Carolina, Charleston, SC, United States; 8 Department of Medical Social Sciences, Northwestern University Feinberg School of Medicine, Chicago, IL, United States

**Keywords:** stroke, aging, cognition, activities of daily living, participation, ecological momentary assessment, smartphone, technology

## Abstract

**Background:**

Stroke is a leading cause of global disability in the aging population, with cognitive impairments playing a significant role. Prior research has shown that subjective cognitive concerns (SCCs) can predict later dementia and serve as an essential indicator for poststroke functional rehabilitation. The use of smartphone-based ecological momentary assessment (EMA) in real-world environments may help us understand how SCCs relate to daily functioning in individuals aging with stroke, thereby guiding cognitive rehabilitation and prevention efforts.

**Objective:**

Our study aimed to use EMA to examine the real-time associations between SCCs and daily activity participation in persons aging with stroke.

**Methods:**

This longitudinal observational study used smartphone-based EMA for real-time assessment of individual cognitive concerns and participation in various daily activities. EMA survey items, including SCCs (concentration and learning) and participation in daily activities (location, company, current activity, and self-appraisals of performance, help needed, satisfaction, and engagement), were collected 5 times per day for 2 weeks. Multilevel models were used to analyze the data.

**Results:**

A total of 202 individuals with mild-to-moderate chronic stroke participated in the study (n=90, 44.6% female; n=89, 44.1% Black; n=182, 90.1% ischemic stroke; mean age 59.7, SD 11.7 years). SCCs were concurrently lower when participants engaged in activities of daily living (ADL; B=−0.04, 95% CI −0.07 to −0.01; *P*=.02), instrumental ADL (B=−0.05, 95% CI −0.07 to −0.02; *P*<.001), cognitively stimulating activities (B=−0.05, 95% CI −0.08 to −0.02; *P*<.001), and social activities (B=−0.05, 95% CI −0.08 to −0.02; *P*=.002); when participants were located in a friend’s home (B=−0.10, 95% CI −0.17 to −0.02; *P*=.001); and when they spent time with family members (B=−0.07, 95% CI −0.10 to −0.04; *P*<.001), friends (B=−0.05, 95% CI −0.10 to −0.01; *P*=.01), and spouse or partners (B=−0.04, 95% CI −0.07 to −0.01; *P*=.02). Conversely, SCCs were higher when participants were in the hospital (B=0.39, 95% CI 0.25‐0.53; *P*<.001). Additionally, greater SCCs were concurrently associated with worse ratings of performance (B=−0.05, 95% CI −0.06 to −0.05; *P*<.001), satisfaction (B=−0.05, 95% CI −0.06 to −0.05; *P*<.001), and activity engagement (B=−0.05, 95% CI −0.06 to −0.04; *P*<.001).

**Conclusions:**

EMA provides an effective means of understanding the links between poststroke cognition and participation in daily activities. Our findings suggest that ADL, instrumental ADL, cognitively demanding activities, and socially engaging activities may lessen cognitive concerns among stroke survivors, implying that clinicians should schedule these activities to help reduce poststroke cognitive issues. Conversely, interventions that enhance cognition may increase participation in these challenging activities. Tracking cognition, everyday activity involvement, and their interactions in real-world settings could ultimately help develop rehabilitation and prevention strategies for individuals at risk of dementia due to stroke.

## Introduction

Stroke is the second most common cause of death and the third leading cause of disability worldwide [1,2], especially in the older population, in which stroke incidence is highest [3]. Alarmingly, stroke incidence in younger age groups (eg, <55 years) has been gradually increasing over the past few decades [4]. Although stroke mortality rates have fallen with advancements in medicine, the burden of disability and dependence on activities of daily living (ADL) after stroke remains substantial. Existing evidence has shown that the incidence of cognitive impairment in the first few weeks following a stroke exceeds 70% [5]. Although many cognitive issues in acute stroke survivors improve within weeks or months after onset [6], longitudinal studies have documented a notable cognitive decline among stroke survivors over long-term follow-up [7], suggesting the importance of investigating the long-term effect of stroke (ie, chronic stroke) and its impact.

Identifying cognitive impairments in research and clinical practice has often relied on objective assessments (eg, neuropsychological testing). In contrast, subjective cognitive concerns (SCCs), defined as whether people self-report cognitive difficulties and, if so, whether and how these problems disturb or annoy them in everyday life, are often neglected [8]. SCCs are commonly reported in people after stroke, with prevalence rates ranging from 28.6% to 90.2%, and these problems often increase over time [9]. The literature suggests that SCCs serve as early indicators of Alzheimer disease and other forms of dementia [10], which are another important source of disability in old age [11,12]. Given that stroke (acute brain tissue damage) itself is a known risk factor for subsequent dementia [13], persons after stroke, in particular, necessitate ongoing monitoring for early detection of cognitive decline. Moreover, cognitive problems prevent stroke survivors from resuming their prestroke roles or achieving independent living [14]. Therefore, monitoring subjective cognitive functioning in everyday life not only helps predict later dementia but also serves as an important indicator of stroke recovery [15].

Research has shown that time spent in passive leisure activities (eg, watching television) is negatively related to cognitive functioning in healthy older adults and increases their risk of developing cognitive impairment in subsequent years [16]. Meanwhile, participation in cognitively, physically, or socially stimulating activities is positively associated with cognitive functioning in healthy older adults and those with medical conditions [17,18]. However, these studies have primarily been conducted in laboratory or clinical settings, often assuming that cognitive characteristics are constant and stable. In fact, cognitive functioning can be influenced by daily life contexts. Furthermore, these studies exploring the link between cognitive difficulties and participation in daily activities have mostly relied on retrospective self-report questionnaires, typically collecting data at one or a few points using cross-sectional or panel designs. These methods may miss subtle changes that happen within and between days and are prone to recall bias. Therefore, it is essential to have a tool that can capture the dynamic interplay of cognition in day-to-day contexts within the study population, such as an ecological momentary assessment (EMA) often implemented via a smartphone. EMA is an intensive longitudinal data collection approach that captures an individual’s daily experiences in real time to minimize recall bias in natural environments, thereby enhancing external validity [19]. Multiple studies have supported the use of EMA in stroke. For example, prior poststroke studies have used EMA to understand real-time relationships between depressed mood and daily activity participation, between somatic and mental symptoms and social interactions [20-22], and to classify poststroke latent cognitive memberships [23]. Although there are studies on these topics, no study has used EMA to identify real-time associations between SCCs and daily activity patterns in stroke survivors. Hence, this study aimed to use EMA to investigate SCCs related to daily activity participation in a sample with mild-to-moderate chronic stroke. We used EMA to evaluate SCCs and the time spent on, as well as participants’ perceptions of, daily activity participation. Our first research question investigated the momentary relationships between SCCs and various factors, including activity types, locations, and interactions with other people. We hypothesized that participants would report higher SCCs when staying at home, being alone, and participating in passive leisure activities. Our second research question examined whether SCCs were momentarily associated with perceived qualities of activities. We hypothesized that SCCs would be higher during activities that participants rated as more difficult, felt less satisfied with, were less engaged in, and required more help.

## Methods

### Participants

Participants were recruited from a local hospital database with active enrollment from October 2018 to January 2021. The inclusion criteria were (1) mild-to-moderate stroke defined by a National Institutes of Health Stroke Scale score of 13 or less at the time of stroke onset, either hemorrhagic or ischemic stroke; (2) at least 3 months after stroke before enrollment; (3) no or mild prestroke disability; and (4) English fluency. People with premorbid neurological or psychiatric disorders, severe communication difficulties, severe apraxia, unilateral visual inattention, or limited visual acuity were excluded from this study.

### Procedures

Participants completed the study protocol, which included an initial laboratory visit, 14-day EMA monitoring, and a post-EMA laboratory visit.

At the initial laboratory visit, all participants provided written informed consent, completed screening to ensure eligibility, and participated in a 20-minute tutorial on using an EMA survey mobile app [24] on either their iPhone or an iPod touch provided by our research team. Participants completed at least 1 practice session to ensure that there were no issues using the app. They were given an EMA manual with hotlines to call should questions arise. As part of the assessment protocol, participants also completed in-laboratory measures, such as the Quality of Life in Neurological Disorders [25] and the Patient-Reported Outcome Measurement Information System [26]. Detailed information on the in-laboratory measures was published elsewhere [20].

The EMA monitoring started within 1 week of the initial visit. Participants completed 5 surveys per day for 14 consecutive days. Surveys were delivered randomly at approximately 2.5-hour intervals per day from 8 AM to 10 PM. Participants had 1 hour to complete each survey, with up to 3 reminders sent before it expired. During the first 2 days and periodically thereafter, the research team contacted participants by phone to address any technical issues or questions they might have while completing the EMA surveys at home.

After EMA monitoring, participants revisited the laboratory to uninstall the EMA app or return the device (if borrowed) and received an honorarium.

### Ethical Considerations

The ethics committees of Washington University (201704024) and Northwestern University (STU00215308) approved this longitudinal observational study. All participants provided written informed consent at the initial laboratory visit. Participants received an honorarium of up to US $125 for their participation in the research.

### Measures

Participants completed a validated EMA survey tailored to the unique needs of stroke survivors (refer to Bui et al [20] for detailed EMA survey questions). Each EMA survey included 23 questions, and participants took about 3 to 5 minutes to complete it. The survey was primarily composed of checkbox- or slider-format questions. At each prompt, participants answered multiple items about their daily activity participation and current levels of SCCs and other poststroke symptoms.

To assess participation in daily activities, participants first reported their current location (eg, home, workplace, and other) and indicated who they spent time with (eg, alone, family members, or others). The subsequent items were customized based on the participant’s reported location, prompting queries about their current activities (eg, working, gardening, etc). On the basis of prior classification models [27], we categorized activities into 7 domains: physical, cognitive, social, ADL, instrumental ADL (IADL), vocational, and passive leisure activities, and further grouped them into total productive and nonproductive activities. After reporting their current activities, participants rated the activity quality in terms of the help needed, performance, satisfaction, and engagement using a 7-point scale, ranging from “no help,” “not well,” “not satisfied,” or “not engaged” to “a lot of help,” “very well,” “very satisfied,” or “very engaged.”

To assess SCCs, participants reported their current levels using the following questions: (1) “I am having trouble concentrating” and (2) “I am having difficulty learning new tasks or instructions,” with a 5-point scale ranging from “not at all” to “very much.” We generated a composite score of SCCs by summing the 2 items, such that high scores indicated more cognitive concerns. These 2 items were moderately correlated (*r*=0.69; *P*<.001) [23]. Our prior research demonstrated a strong association between the EMA-measured SCCs and the Quality of Life in Neurological Disorders cognitive function (*r*=−0.64; *P*<.001) [20].

### Statistical Analysis

We used descriptive statistics to examine the demographics of the study sample. Multilevel models (MLMs) examined the real-time associations between SCCs and participation in daily activities. MLMs treated data on 2 levels; EMA observations were modeled as level 1 (within-person level) nested within individuals modeled as level 2 (between-person level). All statistical tests were 2-sided, with a significance level of *P*<.05.

We conducted models focusing on concurrent associations, including activity type, location, the person participants were interacting with, and quality of activity as predictors of same-survey SCCs. EMA variables were centered to generate person-centered deviation scores. The centered value reflected momentary changes relative to each individual’s weekly average. Centering allows the examination of within-subject and between-subject variances separately [28]. We used PROC MIXED in SAS (version 9.4; SAS Institute) to model both between-subject and within-person variances and account for autocorrelation between adjacent observations. PROC MIXED is robust in handling missing data. Thus, all data were analyzed, and random missingness was assumed [1].

## Results

### Participant Characteristics

**Figure 1.**
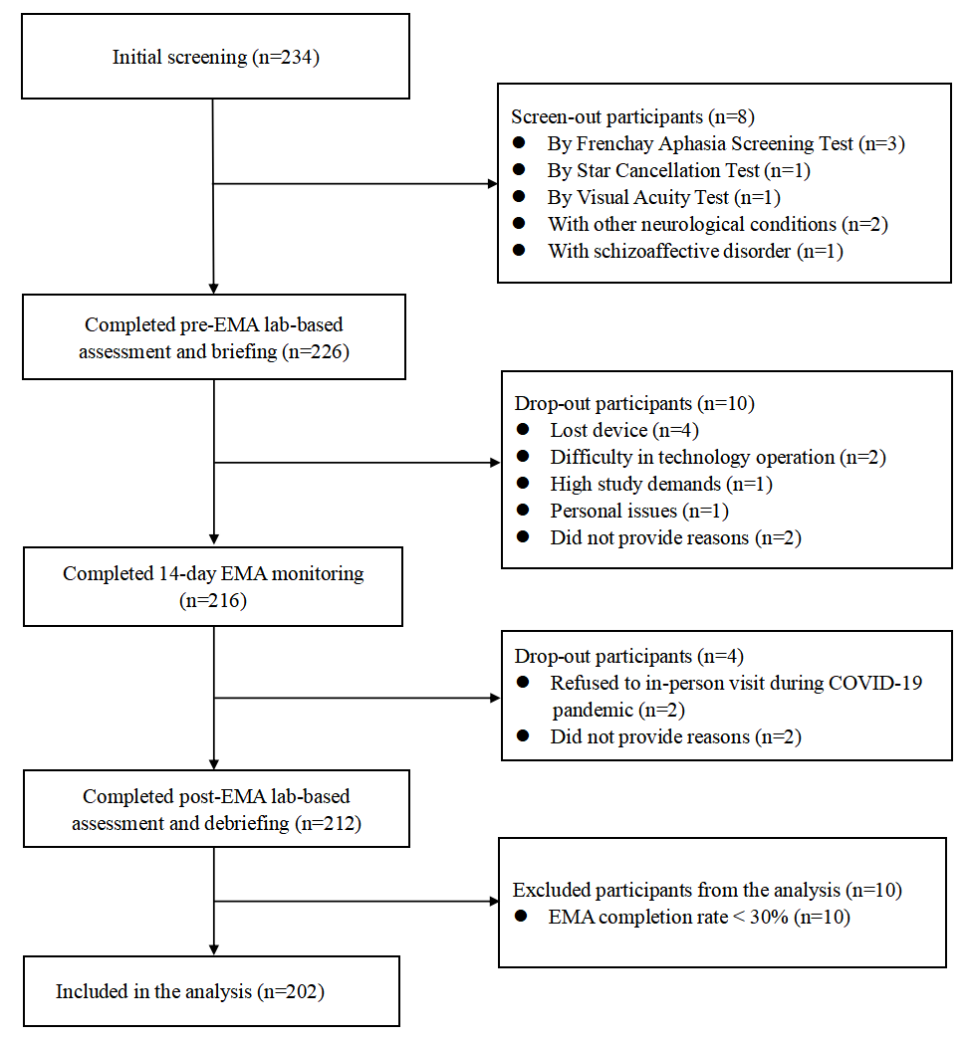
The flow chart of the ecological momentary assessment (EMA) study.

Figure 1 presents the study flow diagram from enrollment to analysis. Of the 212 participants who completed the full study protocol, 10 (4.7%) were excluded because they had an EMA completion rate below 30%. The final sample consisted of 202 (95.3%) stroke survivors (n=90, 44.6% women; mean age 59.7, SD 11.7 years). We observed no significant differences in most demographic and clinical characteristics between the included and excluded participants, except that the included participants had higher global cognitive function (*P*=.02) and more experience with mobile device use (*P*=.02). Table 1 presents the details of participant characteristics. We included a total of 14,140 EMA observations for subsequent analyses.

**Table 1.** Clinicodemographic information of participants.

Variables	Overall (n=212)	Excluded participants (n=10)	Included participants (n=202)	*P* value^a^
Time since stroke (months), mean (SD)	48.31 (34.18)	54.63 (45.96)	48.00 (33.61)	.84
Age (years), mean (SD)	59.85 (11.69)	62.10 (11.45)	59.74 (11.72)	.46
Gender, n (%)				.52
Female	93 (43.9)	3 (30)	90 (44.6)	
Male	119 (56.1)	7 (70)	112 (55.4)	
Race, n (%)				.09
Asian	1 (0.5)	0 (0)	1 (0.5)	
Black	97 (45.8)	8 (80)	89 (44.1)	
White	114 (53.8)	2 (20)	112 (55.4)	
Marital status, n (%)				.08
Married or cohabitating	107 (50.5)	2 (20)	105 (52)	
Separated, divorced, or widowed	66 (31.1)	6 (60)	60 (29.7)	
Single	39 (18.4)	2 (20)	37 (18.3)	
Residential status, n (%)				.06
Alone	51 (24.1)	5 (50)	46 (22.8)	
With others	161 (75.9)	5 (50)	156 (77.2)	
Stroke diagnosis, n (%)				.60
Hemorrhagic	20 (9.4)	0 (0)	20 (9.9)	
Ischemic	192 (90.6)	10 (100)	182 (90.1)	
Stroke side^b^, n (%)				.28
Bilateral	9 (4.2)	0 (0)	9 (4.5)	
Left	84 (39.6)	3 (30)	81 (40.1)	
Right	83 (39.2)	3 (30)	80 (39.6)	
Unknown	35 (16.5)	4 (40)	31 (15.3)	
Premorbid disability (mRS^c^), n (%)				.19
0	177 (83.5)	8 (80)	169 (83.7)	
1	20 (9.4)	0 (0)	20 (9.9)	
2	14 (6.6)	2 (20)	12 (5.9)	
Number of previous strokes, mean (SD)	1.65 (1.76)	1.78 (1.09)	1.65 (1.78)	.23
Stroke severity (NIHSS^d^), mean (SD)	3.23 (3.55)	3.50 (3.17)	3.21 (3.57)	.55
Comorbidity, mean (SD)	4.56 (2.87)	5.11 (2.26)	4.53 (2.90)	.31
Education (years), mean (SD)	14.13 (2.63)	12.80 (2.97)	14.20 (2.60)	.07
Employment status, n (%)				.20
Full time	57 (26.9)	0 (0)	57 (28.2)	
Not employed	125 (59)	9 (90)	116 (57.4)	
Part time	27 (12.7)	1 (10)	26 (12.9)	
Volunteer	3 (1.4)	0 (0)	3 (1.5)	
Global cognition function (MoCA^e^), mean (SD)	25.86 (3.26)	22.10 (5.32)	26.04 (3.02)	.02
Mobile device use experience, n (%)				.02
No	6 (2.9)	2 (22)	4 (2)	
Yes	203 (97.1)	7 (78)	196 (98)	

a Fisher exact test; Wilcoxon rank-sum test.

b n=1 participant missing information.

c mRS: modified Rankin Scale.

d NIHSS: National Institutes of Health Stroke Scale.

e MoCA: Montreal Cognitive Assessment.

### Concurrent Relationships Between SCCs and Daily Activity Participation

**Figure 2.**
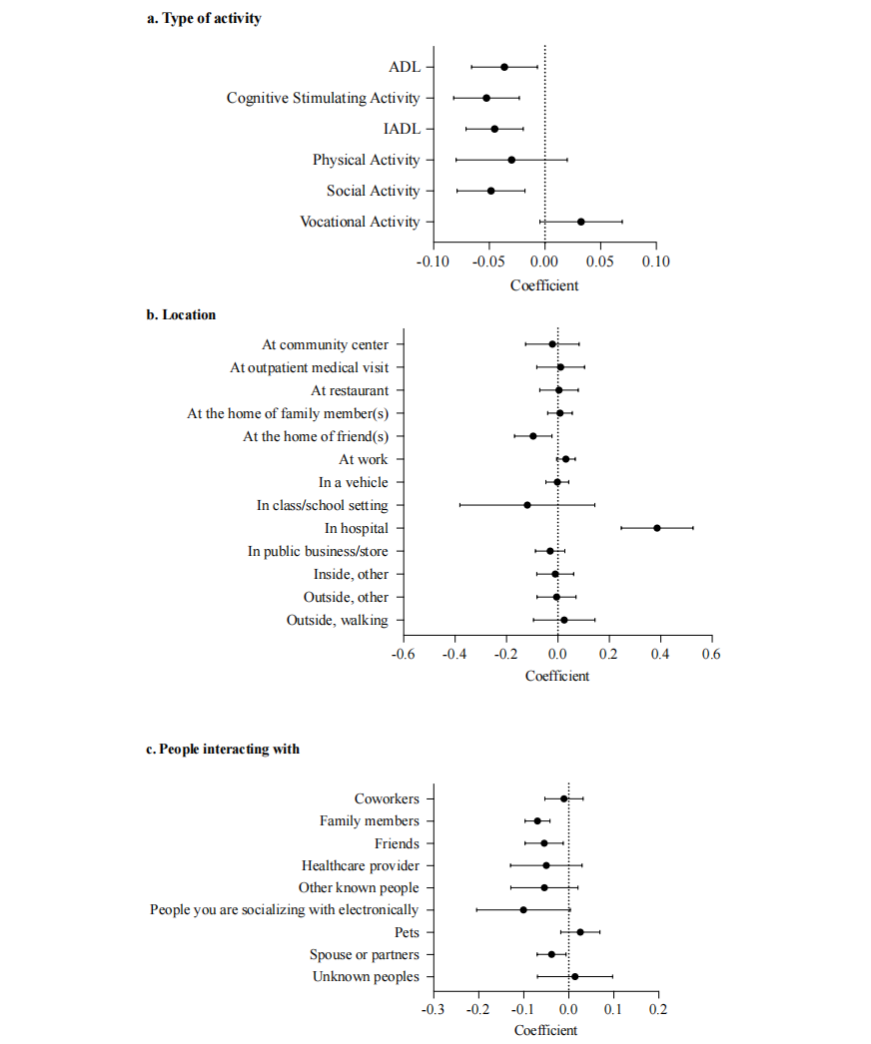
Concurrent relationships of subjective cognitive concerns with daily activity participation are as follows: (A) type of activity (reference: passive leisure activity), (B) location (reference: being at home), and (C) person interacting with (reference: being alone). ADL: activities of daily living; IADL: instrumental activities of daily living.

Descriptive statistics on the daily activity participation, measured by EMA, were reported in our previous publication [20]. By testing the activity type from a total of 11,580 EMA observations, MLM results showed that lower SCCs were significantly associated with more time spent performing ADL (B=−0.04, 95% CI −0.07 to −0.01; *P=*.02), IADL (B*=*−0.05, 95% CI −0.07 to −0.02; *P<*.001), cognitively stimulating activity (B*=*−0.05, 95% CI −0.08 to −0.02; *P<*.001), and social activity (B*=*−0.05, 95% CI −0.08 to −0.02; *P=*.002) relative to passive leisure activity as the reference. We also found no significant relationships between SCCs and physical or vocational activity (Figure 2A and Table 2).

**Table 2.** Results of multilevel models examining concurrent relationships between subjective cognitive concerns and daily activity participation.

Variables	EMA^a^ surveys, n (%)	B (SE; 95% CI)	*P* value
Type of activity (n=11,580)			
Intercept	—^b^	0.38 (0.04; 0.30 to 0.46)	<.001
Activities of daily living	1419 (12.3)	−0.04 (0.02; −0.07 to −0.01)	.02
Cognitive stimulating activity	1467 (12.7)	−0.05 (0.02; −0.08 to −0.02)	<.001
Instrumental activities of daily living	2505 (21.6)	−0.05 (0.01; −0.07 to −0.02)	<.001
Physical activity	415 (3.6)	−0.03 (0.03; −0.08 to 0.02)	.24
Social activity	1523 (13.2)	−0.05 (0.02; −0.08 to −0.02)	.002
Vocational activity	993 (8.6)	0.03 (0.02; −0.00 to 0.07)	.09
Passive leisure activity	3258 (28.1)	Referent	—
Location (n=11,642)			
Intercept	—	0.35 (0.04; 0.27 to 0.43)	<.001
At community center	93 (0.8)	−0.02 (0.05; −0.13 to 0.08)	.69
At outpatient medical visit	103 (0.9)	0.01 (0.05; −0.08 to 0.10)	.82
At restaurant	186 (1.6)	0.00 (0.04; −0.07 to 0.08)	.91
At the home of family members	552 (4.7)	0.01 (0.02; −0.04 to 0.06)	.74
At the home of friends	181 (1.6)	−0.10 (0.04; −0.17 to −0.02)	.01
At work	1002 (8.6)	0.03 (0.02; −0.01 to 0.07)	.09
In a vehicle	439 (3.8)	0.00 (0.02; −0.05 to 0.04)	.92
In class or school setting	18 (0.2)	−0.12 (0.13; −0.38 to 0.14)	.37
In hospital	57 (0.5)	0.39 (0.07; 0.25 to 0.53)	<.001
In public business or store	293 (2.5)	−0.03 (0.03; −0.09 to 0.03)	.30
Inside, other	187 (1.6)	−0.01 (0.04; −0.08 to 0.06)	.78
Outside, other	177 (1.5)	−0.01 (0.04; −0.08 to 0.07)	.89
Outside, walking	56 (0.5)	0.02 (0.06; −0.10 to 0.14)	.69
At my home	8298 (71)	Referent	—
Person interacting with (n=8860)			
Intercept	—	0.38 (0.04; 0.30 to 0.46)	<.001
Coworkers	581 (5.1)	−0.01 (0.02; −0.05 to 0.03)	.63
Family members	1940 (17)	−0.07 (0.01; −0.10 to −0.04)	<.001
Friends	441 (3.9)	−0.05 (0.02; −0.10 to −0.01)	.01
Health care provider	118 (1)	−0.05 (0.04; −0.13 to 0.03)	.22
Other known people	128 (1.1)	−0.05 (0.04; −0.13 to 0.02)	.16
People you are socializing with electronically	64 (0.6)	−0.10 (0.05; −0.20 to 0.00)	.06
Pets	861 (7.6)	0.03 (0.02; −0.02 to 0.07)	.24
Spouse or partners	1518 (13.3)	−0.04 (0.02; −0.07 to −0.01)	.02
Unknown people	91 (0.9)	0.01 (0.04; −0.07 to 0.10)	.74
Alone	3118 (27.4)	Referent	—

a EMA: ecological momentary assessment.

b Not applicable.

By testing the location from a total of 11,642 EMA observations, MLM results revealed that participants reported lower SCCs at the home of friends (B*=*−0.10, 95% CI −0.17 to −0.02; *P=*.001) but higher SCCs in the hospital, including inpatient and outpatient visits (B*=*0.39, 95% CI 0.25‐0.53; *P<*.001) compared with being at home. However, the relationships between SCCs and other locations (eg, community centers and workplaces) were not robust enough to achieve significance compared with being at home (Figure 2B and Table 2).

By testing the person participants were interacting with from a total of 8860 EMA observations, MLM results indicated that participants had lower SCCs when they spent time with their family members (B*=*−0.07, 95% CI −0.10 to −0.04; *P<*.001), friends (B*=*−0.05, 95% CI −0.10 to −0.01; *P=*.01), and spouse or partners (B*=*−0.04, 95% CI −0.07 to −0.01; *P=*.02) when compared with being alone. We found no significant relationships between SCCs and interactions with coworkers, health care providers, and others (Figure 2C and Table 2).

### Concurrent Relationships Between SCCs and Activity Qualities

By testing the activity quality from a total of 11,580 EMA observations, MLM results revealed significant associations between greater perceived performance (B=−0.05, 95% CI −0.06 to −0.05; *P<*.001), satisfaction (B*=*−0.05, 95% CI −0.06 to −0.05; *P<*.001), and engagement with lower SCCs (B=−0.05, 95% CI −0.06 to −0.04; *P<*.001); however, perceived help did not show a statistically significant relationship with SCCs (B*=*0.00, 95% CI −0.01 to 0.00; *P=*.31; Table 3).

**Table 3.** Results of multilevel models examining concurrent relationships between subjective cognitive concerns and activity quality.

Variables	B (SE; 95% CI)	*P* value
Help from someone while doing activities		
Intercept	0.36 (0.05; 0.25 to 0.46)	<.001
Help score (between-person)	−0.01 (0.03; −0.08 to 0.05)	.63
Help score (within-person)	0.00 (0.00; −0.01 to 0.00)	.31
Performance of activities		
Intercept	1.86 (0.17; 1.52 to 2.20)	<.001
Performance score (between-person)	−0.30 (0.03; −0.36 to −0.23)	<.001
Performance score (within-person)	−0.05 (0.00; −0.06 to −0.05)	<.001
Satisfaction with doing activities		
Intercept	1.83 (0.17: 1.51 to 2.16)	<.001
Satisfaction score (between-person)	−0.29 (0.03: −0.36 to −0.23)	<.001
Satisfaction score (within-person)	−0.05 (0.00: −0.06 to −0.05)	<.001
Engagement in activities		
Intercept	1.57 (0.16: 1.25 to 1.88)	<.001
Engagement score (between-person)	−0.25 (0.03: −0.31 to −0.19)	<.001
Engagement score (within-person)	−0.05 (0.00: −0.06 to −0.04)	<.001

## Discussion

### Principal Findings

This study provides the first in-the-moment examination of the relationships between SCCs and daily activity participation reported on an EMA survey in stroke survivors in naturalistic environments. With respect to our first hypothesis, the results indicated that after stroke, SCCs were not static but were experienced dynamically in daily life. Specifically, SCCs were significantly lower when performing ADL or IADL, engaging in cognitively stimulating activity, and attending social activities than when engaging in passive leisure activities among stroke survivors. Our findings align with a previous real-time assessment study by Moore et al [29], which found greater cognition and multitasking abilities were associated with less time spent in passive leisure activities among older adults living with HIV. Unexpectedly, despite existing evidence consistently indicating that cognitive impairments are linked to high levels of sedentary behavior and low levels of physical activity [30], such a relationship was not observed in this EMA study. Indeed, poststroke cognitive impairment may influence participation in physical activity [31], but the relationship is complex and may not always be direct. Other factors, such as physical limitations, psychological issues, and social support, may mediate how cognitive impairment affects physical activity participation in stroke survivors [32,33]. Further research is necessary to identify these factors as moderators or mediators and to unravel the relationship between after stroke SCCs and physical activity participation in real time.

The results revealed that SCCs were significantly lower among stroke survivors when they were at a friend’s home, while higher SCCs were observed in the hospital compared with being at home. This difference may be attributed to psychological factors. As we know, people likely feel more comfortable and relaxed in the familiar surroundings of a friend’s home. In contrast, an unanticipated visit to the hospital may impose additional psychological burdens, such as stress and anxiety, leading to increased concerns of cognitive difficulties [34].

This argument can be extended to another significant finding: stroke survivors exhibited lower SCCs when spending time with family members, friends, or partners compared with when they were alone. Individuals may be more susceptible to psychological challenges when alone than when surrounded by their families and friends [35]. Other factors, such as a lack of social support and limited mobility, may further contribute to heightened SCCs in stroke survivors when they are alone [36].

It is important to recognize that the relationship between SCCs and participation in daily activities may be bidirectional. Cognitive difficulties experienced by stroke survivors can be triggered by certain activities, but these difficulties can also affect their willingness to engage in and choice of activities. For example, stroke survivors who reported higher levels of SCCs were more likely to avoid cognitively demanding activities or social interactions beyond family members and friends. Because the time-lagged relationships between SCCs and daily activity participation, such as responses from a previous EMA survey predicting responses on the current survey, have not been examined, the causal direction remains unclear. Future research could use time-lagged models to investigate these cause-and-effect relationships.

Regarding the second hypothesis, our results showed that SCCs were significantly lower when stroke survivors rated their performance, satisfaction, and engagement in daily activities as higher. It is consistent with previous research [37] indicating that cognitive impairment after stroke is closely linked to poorer performance in daily activities, suggesting that how stroke survivors rate their everyday functional limitations is closely related to their ratings of SCCs. Contrary to our hypothesis, seeking help from others was not significantly associated with SCCs. A prior study on help-seeking behaviors found that many community-dwelling stroke survivors with cognitive difficulties often hesitate to seek assistance from health care professionals. This reluctance can stem from fears of receiving a dementia diagnosis, denial of symptoms as part of “natural” aging, and limited access to care in their community [38]. These results indicate a need for screening and risk assessment of stroke survivors to prevent more severe cognitive impairments or the onset of dementia within the health care system. Future interventions should focus on ensuring that stroke survivors and their caregivers have access to support and information, as well as facilitating referrals back to specialized services if cognitive symptoms arise or persist.

Despite the contributions of this study, several limitations should be acknowledged. First, regarding our EMA survey, in-the-moment SCCs were assessed using only 2 single items. These 2 items may not provide a comprehensive measurement of cognitive functioning compared with a multi-item survey. Future studies should incorporate additional items that assess diverse cognitive domains, such as processing speed, working memory, executive functioning, and receptive and expressive language, to determine whether all types of cognitive concerns are equally significant for participation in daily activities. Furthermore, the 2 items in our study were moderately correlated, suggesting that these 2 cognitive domains may be differently associated with the daily activity predictors included in the study. Because we did not provide an option for participants to respond “not applicable” in this study, we are unable to determine whether the absence of subjective reports of cognitive difficulty reflects a lack of situational demands related to attention or learning, rather than preserved disability. This further underscores the importance of treating the association between perceived difficulties in each cognitive domain and daily activities in considerable detail to achieve a more accurate understanding of the dynamics of cognition and activity participation.

Second, two-thirds of our participants were community-dwelling individuals with neurologically mild stroke, which limits the generalizability of our results to a broader stroke population. Notably, we found that the cognitive function of participants who dropped out was significantly worse than that of participants who were included in the study, suggesting that the EMA method may not be particularly well-suited for stroke populations with more severe cognitive impairment. Similar concerns also apply to populations that lack experience with mobile devices, which significantly contributed to the dropout rate in this EMA study. Third, there may be other confounding variables affecting SCCs in stroke survivors that were not accounted for in this study, such as negative affect [21]. Future research should investigate the specific risk factors associated with developing SCCs after stroke. Additionally, while the use of EMA is a strength of this study, we only monitored participants for 14 days. It remains unclear whether these findings would hold true over a longer monitoring period.

### Conclusions

The findings of this study, which used dynamic data collection methods in natural settings and involved a large sample size, support the idea that SCCs fluctuate among stroke survivors in their daily lives. Furthermore, our study highlights the importance of ongoing monitoring of cognitive concerns and daily activity functioning. This monitoring enables us to identify cognitive and functional challenges faced by individuals aging with stroke. Monitoring these factors and their dynamics in real-world contexts may ultimately help identify rehabilitation and prevention strategies for individuals at risk of dementia due to stroke.

## Abbreviations

ADL: activities of daily living
EMA: ecological momentary assessment
IADL: instrumental activities of daily living
MLM: multilevel model
SCC: subjective cognitive concern
